# Meta-analysis of the diagnostic performance of stress perfusion cardiovascular magnetic resonance for detection of coronary artery disease

**DOI:** 10.1186/1532-429X-12-29

**Published:** 2010-05-19

**Authors:** Michèle Hamon, Georges Fau, Guillaume Née, Javed Ehtisham, Rémy Morello, Martial Hamon

**Affiliations:** 1 Department of Radiology, University Hospital of Caen, France; 2 INSERM 919, Cyceron, Caen, France; 3 Greyc Laboratory, CNRS UMR 6072, Caen, France; 4 Department of Cardiology, University Hospital of Caen, France; 5 Department of Statistics, University Hospital of Caen, France; 6 INSERM 744, Institut Pasteur de Lille, France

## Abstract

**Aim:**

Evaluation of the diagnostic accuracy of stress perfusion cardiovascular magnetic resonance for the diagnosis of significant obstructive coronary artery disease (CAD) through meta-analysis of the available data.

**Methodology:**

Original articles in any language published before July 2009 were selected from available databases (MEDLINE, Cochrane Library and BioMedCentral) using the combined search terms of magnetic resonance, perfusion, and coronary angiography; with the exploded term coronary artery disease. Statistical analysis was only performed on studies that: (1) used a [greater than or equal to] 1.5 Tesla MR scanner; (2) employed invasive coronary angiography as the reference standard for diagnosing significant obstructive CAD, defined as a [greater than or equal to] 50% diameter stenosis; and (3) provided sufficient data to permit analysis.

**Results:**

From the 263 citations identified, 55 relevant original articles were selected. Only 35 fulfilled all of the inclusion criteria, and of these 26 presented data on patient-based analysis. The overall patient-based analysis demonstrated a sensitivity of 89% (95% CI: 88-91%), and a specificity of 80% (95% CI: 78-83%). Adenosine stress perfusion CMR had better sensitivity than with dipyridamole (90% (88-92%) versus 86% (80-90%), P = 0.022), and a tendency to a better specificity (81% (78-84%) versus 77% (71-82%), P = 0.065).

**Conclusion:**

Stress perfusion CMR is highly sensitive for detection of CAD but its specificity remains moderate.

## Introduction

Perfusion cardiovascular magnetic resonance (CMR) is an emerging technique for the detection of coronary artery disease (CAD). The technique is attractive because of its non-invasive nature and safe characteristics, and might potentially play a major role in future diagnosis and risk stratification guidelines for patients with suspected CAD. Several small studies have evaluated the diagnostic performance of stress perfusion CMR and some of those have been included in a previous meta-analysis [1]. In the current study we provide a comprehensive and contemporary meta-analysis of its diagnostic accuracy compared with an invasive coronary angiography (CA) used as a reference standard.

## Methods

### Search strategy

Using the combined medical subject headings (MeSH) of *magnetic resonance, perfusion*, and *coronary angiography*, with the exploded terms *coronary artery disease*; the MEDLINE, Cochrane Library and BioMedCentral databases were searched independently by two investigators (MH, GF) for all publications, in any language, before July 2009. In addition, the published reference lists of these articles were systematically searched.

### Study eligibility

The search results were collated by the same two investigators (MH, GF), and duplicate or overlapping papers removed. Studies were eligible if: [1] stress perfusion CMR was used as a diagnostic test for significant obstructive CAD; [2] conventional invasive CA was used as the reference standard for diagnosing significant obstructive CAD, defined as a ≥50% diameter stenosis; and [3] the absolute numbers of true positive (TP), false positive (FP), true negative (TN), and false negative (FN) were reported, or could be derived. Studies were excluded if they were performed with a 0.5 or 1 Tesla MR scanner, if they included less than 10 patients, and if only abstracts from scientific meetings were published as the data provided may either be not sufficiently detailed or finalized. Any disagreements on eligibility were resolved by discussion and consensus between the two investigators.

### Data extraction and quality assessment

Data extraction was performed independently by the two investigators (MH, GF) for each study. The following fields were recorded: study population size; gender distribution; mean age and standard deviation; number of patients with documented CAD; prevalence of CAD; relative timing of the two imaging procedures; the degree of blinding in interpretation of test results (both to the patient's clinical context and the results of the other imaging modality); type and brand of MR machine used; the type of perfusion stressor (adenosine, nicorandil, dipyridamole), and the number of side effects; the dose and injection rate of Gadolinium administrated; and the modality of MR image analysis (visual, or semi-quantitative). Any discrepancies were resolved by discussion and consensus between the two investigators. Where available, data was recorded separately at the level of coronary territories and coronary arteries. The study quality conformed to the Quality Assessment of Studies of Diagnostic Accuracy included in Systematic Reviews guidelines [2]. In one study, for which patients were evaluated both with 1.5 and 3T CMR, we used 1.5 T data in the meta-analysis. For the studies where analysis was performed with both 50% and 70% coronary stenosis definitions, we included results with the 70% definition in the pooled reported sensitivity and specificity.

### Data synthesis and statistical analysis

Data analysis was performed at the level of the patient, the coronary territory and the coronary artery. Sensitivity and specificity were calculated using the TP, TN, FP, and FN rates [3,4]. From these were calculated the likelihood ratios, which express how much the odds of significant obstructive CAD change in the presence of either an abnormal stress perfusion CMR (positive likelihood ratio: PLR = sensitivity/(1- specificity)), or a normal stress perfusion CMR (negative likelihood ratio: NLR = (1- sensitivity)/specificity). Finally, the ratio of the PLR to the NLR was used to calculate the diagnostic odds ratio (DOR), which estimates how much greater the odds of having significant obstructive CAD are for patients with a positive test result compared with a negative one.

All these measures of diagnostic accuracy were calculated for each individual study and reported as point estimates with 95% confidence intervals. They were then combined using a random-effects model and each point estimate weighted by the inverse of the sum of its variance and the between-study variance. We also assessed between-study statistical heterogeneity using the Cochran Q chi-square tests (cut off for statistical significance *P *≤ .10). Since diagnostic parameters are, by definition, interdependent, independent weighting may sometimes give spurious results and provide biased estimates; to overcome the interdependence problem, we computed the weighted symmetric summary receiver operating characteristic curve, with pertinent areas under the curve, using the Moses-Shapiro-Littenberg method [5-7]. All statistical calculations were performed with SPSS 14.0 (SPSS, Chicago, IL) and Meta-DiSc [8], and significance testing was at the two-tailed 0.05 level [9].

## Results

**Figure 1:**
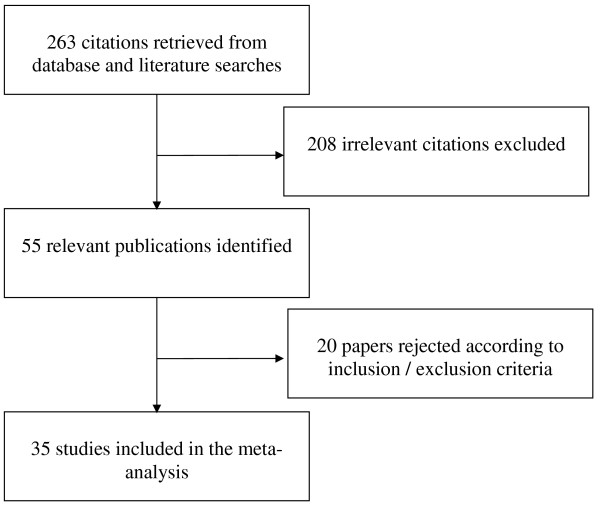
**Flow diagram of the reviewing process**.

Database and literature searches retrieved 263 citations, amongst which 55 relevant publications were identified (Figure 1). Further scrutiny led 20 papers to be rejected either because of overlapping data, or exclusion criteria were met (employed 0.5 or 1 T CMR, or inclusion criteria were absent (impossible to find or calculate absolute figures from presented data). Therefore, 35 studies were finally included in the meta-analysis [10-44], all of which had been published between 2000 and 2009. Study and population characteristics are summarized in Table 1, and the results of the pooled analyses are summarized in Table 2. Dose of contrast Gadolinium administrated range from 0.025 to 0.15 mmol/kg, with an injection rate varying from 3 to 10 mL/s. Quality assessments for all included studies are shown in Table 3. The 35 papers eligible for the analyses comprised 2,456 patients, and of the 2,154 patients for whom gender and the age were specified, 1,481 were males (68.7%) and the mean age was 61.3 years.

**Table 1:** Characteristics of included studies

Authors	Year	Brand	Tesla	Patients (n)	Excluded (n)	Male (%)	Mean Age (SD)	Prevalence (% per patient)	Coronary Stenosis (%)	Stressor*	Side Effects ** (n)	Data assessment
*Al Saadi, (10)*	2000	Philips	1.5	40	6	-	-	100	≥ 75	D	0	1/2 Quantitative
*Schwitter (11)*	2001	GE	1.5	48	1	83	59(-)	79	≥ 50	D	0	1/2 Quantitative
*Ibrahim, (12)*	2002	Philips	1.5	25	0	76	63(13)	100	> 75	A	-	1/2 Quantitative
*Sensky (13)*	2002	Siemens	1.5	30	0	90	62(-)	100	> 50	A	0	Visual
*Chiu, (14)*	2003	Siemens	1.5	13	0	54	68(-)	92	> 50	A	0	Visual
*Doyle (15)*	2003	Philips	1.5	229	45	0	59(11)	14	≥ 70	D	-	1/2 Quantitative
*Ishida (16)*	2003	GE	1.5	104	0	78	66(12)	74	> 70	D	0	Visual
*Nagel (17)*	2003	Philips	1.5	90	6	81	63(8)	51	≥ 75	A	2	1/2 Quantitative
*Bunce (18)*	2004	Picker	1.5	35	0	77	56(12)	49	≥ 50	A	0	1/2 Quantitative
*Giang (19)*	2004	GE	1.5	94	14	69	58(-)	65	≥ 50	A	0	1/2 Quantitative
*Kawase (20)*	2004	Philips	1.5	50	0	58	66(12)	66	≥ 70	N	0	Visual
*Paetsch (21)*	2004	Philips	1.5	79	0	66	61(9)	67	> 50	A	0	Visual
*Plein (22)*	2004	Philips	1.5	72	4	79	57(11)	82	≥ 70	A	1	Visual
*Takase (23)*	2004	GE	1.5	102	-	83	66(9)	74	> 50	D	-	Visual
*Thiele (24)*	2004	Philips	1.5	20	0	-	64(8)	90	≥ 70	A	0	1/2 Quantitative
*Okuda (25)*	2005	GE	1.5	33	0	88	60(-)	97	≥ 75	D	0	Visual
*Plein (26)*	2005	Philips	1.5	92	10	74	58(-)	72	> 70	A	0	1/2 Quantitative
*Sakuma (27)*	2005	Siemens	1.5	40	0	70	65(9)	52	> 70	D	0	Visual
*Cury (28)*	2006	GE	1.5	47	1	81	63(5)	65	≥ 70	D	-	Visual
*Klem (29)*	2006	Siemens	1.5	100	8	49	58(11)	40	>50/≥ 70	A	1	Visual
*Pilz (30)*	2006	GE	1.5	176	5	63	62(12)	66	> 70	A	2	Visual
*Rieber (31)*	2006	Siemens	1.5	50	7	88	61(8)	67	> 50	A	0	1/2 Quantitative
*Cheng (32)*	2007	Siemens	1.5/3	65	4	75	64(8)	66	≥ 50	A	1	Visual
*Costa (33)*	2007	Siemens	1.5	37	7	53	65(11)	97	> 50/> 70	A	0	1/2 Quantitative
*Greenwood (34)*	2007	Philips	1.5	35	0	89	55(-)	83	≥ 70	A	0	Visual
*Kühl (35)*	2007	Philips	1.5	20	1	68	64(13)	100	≥ 50	A	0	1/2 Quantitative
*Merkle (36)*	2007	Philips	1.5	228	0	79	61(11)	75	> 50/> 70	A	0	Visual
*Seeger (37)*	2007	Siemens	1.5	51	0	86	65(9)	74	> 70	A	0	1/2 Quantitative
*Gebker (38)*	2008	Philips	3	101	3	70	62(8)	69	≥ 50	A	2	Visual
*Meyer (39)*	2008	Philips	3	60	0	63	59(10)	60	≥ 70	A	0	Visual
*Pilz (40)*	2008	GE	1.5	22	0	64	66(12)	33	≥ 70	A	0	Visual
*Klein (41)*	2008	Philips	1.5	54	5	65	60(10)	47	≥ 50	A	2	Visual
*Klem (42)*	2008	Siemens	1.5	147	11	0	63(11)	27	≥ 70	A	0	Visual
*Thomas (43)*	2008	Philips	3	60	0	68	-	47	≥ 50	A	0	Visual
*Burgstahler (44)*	2008	Philips	1.5	23	3	65	68(12)	40	≥ 70	A	0	Visual

* Stressor: A (Adenosine); D (Dypirydamole); N (Nicorandil) ** n: significant side effects, which led to stop the MR exam.

**Table 2:** Pooled summary results

Studies	N studies	N	Sensitivity	Specificity	Positive Likelihood ratio	Negative likelihood ratio	Diagnostic odds ratio
**Per Patient analysis (all)**	26	2125 Patients	89% (88-91)	80% (78-83)	4.18 (3.31-5.27)	0.15 (0.11-0.20)	33.65 (22.09-51.27)

Adenosine stressor	20	1658 Patients	90% (88-92)	81% (78-84)	4.47 (3.39-5.88)	0.14 (0.11-0.18)	37.17 (25.16-54.91)

Dipyridamole stressor	5	417 Patients	86% (80-90)	77% (71-82)	2.97 (2.16-4.09)	0.20 (0.09-0.45)	17.03 (5.56 - 52.18)

Visual assessment	20	1624 Patients	91% (89-93)	79% (76-83)	4.08 (3.15-5.29)	0.13 (0.10-0.17)	36.79 (23.90-56.63)

Semi-quant. assessment	6	501 Patients	82%(77-87)	82% (77-86)	4.88 (2.62-9.09)	0.22 (0.13-0.37)	25.44 (8.90-72.70)

**Per Territory analysis**	17	2709 Territories	82% (79-84)	84% (82-85)	4.90(3.66-6.55)	0.23 (0.20-0.27)	23.23 (18.33-29.45)

**Per Artery analysis**							

LAD	8	662 Arteries	83%(78-88)	83%(79-86)	4.37(2.96-6.44)	0.22 (0.16-0.31)	21.42 (10.94-41.94)

CX	8	672 Arteries	76%(70-82)	87%(84-90)	5.74(3.94-8.35)	0.30 (0.23-0.38)	22.25 (14.09-35.10)

RCA	8	657 Arteries	78%(71-84)	87% (83-90)	5.58 (3.74-8.32)	0.29 (0.21-0.38)	23.07 (14.55-36.57)

**Table 3:** Quality assessment (QUADAS)

Study	Item 1	Item 2	Item 3	Item 4	Item 5	Item 6	Item 7	Item 8	Item 9	Item 10	Item 11	Item 12	Item 13	Item 14
*Al Saadi, 2000 (10)*	no	yes	yes	unclear	yes	yes	yes	no	no	unclear	unclear	no	yes	yes
*Schwitter, 2001 (11)*	yes	yes	yes	yes	yes	yes	yes	yes	Yes	yes	yes	yes	yes	yes
*Ibrahim, 2002 (12)*	yes	yes	yes	unclear	yes	yes	yes	yes	Yes	unclear	unclear	yes	yes	yes
*Sensky, 2002 (13)*	yes	yes	yes	unclear	yes	yes	yes	yes	Yes	yes	yes	yes	yes	yes
*Chiu, 2003 (14)*	yes	yes	yes	yes	yes	yes	yes	yes	Yes	yes	yes	yes	yes	yes
*Doyle, 2003 (15)*	yes	yes	yes	unclear	yes	yes	yes	yes	Yes	no	no	yes	yes	yes
*Ishida,2003 (16)*	yes	yes	yes	yes	yes	yes	yes	yes	Yes	yes	yes	yes	yes	yes
*Nagel, 2003 (17)*	yes	yes	yes	yes	yes	yes	yes	yes	Yes	yes	yes	yes	yes	yes
*Bunce, 2004 (18)*	yes	yes	yes	yes	yes	yes	yes	yes	Yes	yes	yes	yes	no	yes
*Giang, 2004 (19)*	yes	yes	yes	yes	yes	yes	yes	yes	Yes	yes	yes	yes	yes	yes
*Kawase, 2004 (20)*	yes	yes	yes	yes	yes	yes	yes	yes	Yes	yes	yes	yes	yes	yes
*Paetsch, 2004 (21)*	yes	yes	yes	unclear	yes	yes	yes	yes	Yes	yes	yes	yes	unclear	unclear
*Plein, 2004 (22)*	yes	yes	yes	yes	yes	yes	yes	yes	Yes	yes	yes	yes	yes	yes
*Takase, 2004 (23)*	yes	yes	yes	yes	yes	yes	yes	yes	Yes	yes	yes	yes	no	unclear
*Thiele,2004 (24)*	yes	yes	yes	no	yes	yes	yes	yes	Yes	yes	yes	yes	yes	yes
*Okuda,2005 (25)*	yes	yes	yes	yes	yes	yes	yes	yes	Yes	yes	yes	yes	yes	yes
*Plein, 2005 (26)*	yes	yes	yes	yes	yes	yes	yes	yes	Yes	yes	yes	yes	yes	yes
*Sakuma,2005 (27)*	yes	yes	yes	yes	yes	yes	yes	yes	Yes	yes	yes	yes	yes	yes
*Cury, 2006 (28)*	yes	yes	yes	yes	yes	yes	yes	yes	Yes	yes	yes	yes	yes	yes
*Klem, 2006 (29)*	yes	yes	yes	yes	yes	yes	yes	yes	Yes	yes	yes	yes	yes	yes
*Pliz, 2006 (30)*	yes	yes	yes	unclear	yes	yes	yes	yes	Yes	yes	yes	yes	yes	yes
*Rieber, 2006 (31)*	yes	yes	yes	yes	yes	yes	yes	yes	Yes	yes	yes	yes	yes	yes
*Cheng, 2007 (32)*	yes	yes	yes	yes	yes	yes	yes	yes	Yes	yes	yes	yes	yes	yes
*Costa,2007 (33)*	yes	yes	yes	yes	yes	yes	yes	yes	Yes	yes	yes	yes	yes	yes
*Greenwood, 2007 (34)*	no	yes	yes	yes	yes	yes	yes	yes	Yes	yes	yes	yes	yes	yes
*Kuhl, 2007 (35)*	yes	yes	yes	yes	yes	yes	yes	yes	Yes	yes	yes	yes	yes	yes
*Merkle, 2007 (36)*	yes	yes	yes	yes	yes	yes	yes	yes	Yes	yes	yes	yes	yes	yes
*Seeger, 2007 (37)*	yes	yes	yes	yes	yes	yes	yes	yes	Yes	yes	yes	yes	yes	yes
*Gebker, 2008 (38)*	yes	yes	yes	yes	yes	yes	yes	yes	Yes	yes	yes	yes	yes	yes
*Meyer, 2008 (39)*	yes	yes	yes	yes	yes	yes	yes	yes	Yes	yes	yes	yes	unclear	unclear
*Pilz, 2008 (40)*	yes	yes	yes	yes	yes	yes	yes	yes	Yes	yes	yes	yes	yes	yes
*Klein,2008 (41)*	yes	yes	yes	yes	yes	yes	yes	yes	Yes	yes	yes	yes	yes	yes
*Klem, 2008 (42)*	no	yes	yes	yes	yes	yes	yes	yes	Yes	yes	yes	yes	yes	yes
*Thomas, 2008 (43)*	yes	yes	yes	unclear	yes	yes	yes	yes	Yes	yes	yes	yes	yes	unclear
*Burgstahler,2008 (44)*	yes	yes	yes	unclear	yes	yes	yes	yes	Yes	unclear	unclear	yes	yes	yes

Item 1: was the spectrum of patients representative of the patients who will receive the test in practice?; Item 2: were selection criteria clearly described?; Item 3: is the reference standard likely to correctly classify the target condition?; Item 4: is the time period between reference and standard and index test short enough to be reasonably sure that the target condition did not change between the two tests?; Item 5: did the whole sample or a random selection of the sample, receive verification using a reference standard of diagnosis?; Item 6: did patients receive the same reference standard regardless of the index test results?; Item 7: was the reference standard independent of the index test (i.e. the index test did not form part of the reference standard); Item 8: was the execution of the index test described in the sufficient detail to permit replication of the test; Item 9: was the execution of the reference standard described in the sufficient detail to permit its replication?; Item 10: were the index test results interpreted without knowledge of the results of the reference standard?; Item 11: were the reference standard results interpreted without knowledge of the results of the index test?; Item 12: were the same clinical data available when test results were interpreted as would be available when the test is used in practice?; Item 13: were uninterpretable/intermediate test results reported?; Item 14: were withdrawals from the study explained.

### Diagnostic performance of stress perfusion CMR: Patient-based analysis

**Figure 2:**
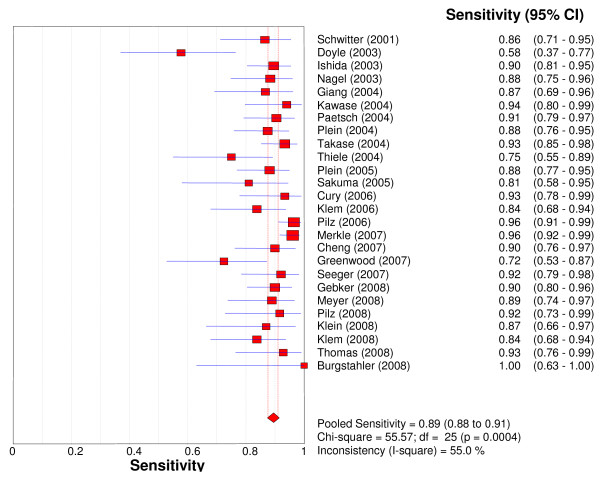
**Forest plot of patient-level sensitivity of stress perfusion CMR, compared with coronary angiography.**.

**Figure 3:**
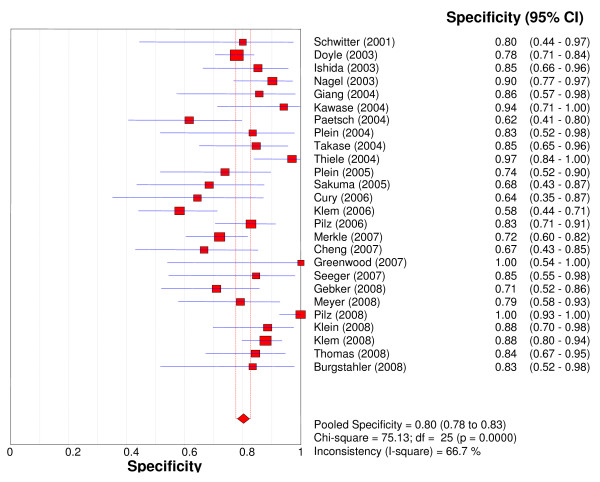
**Forest plot of patient-level specificity of stress perfusion CMR, compared with coronary angiography**.

**Figure 4:**
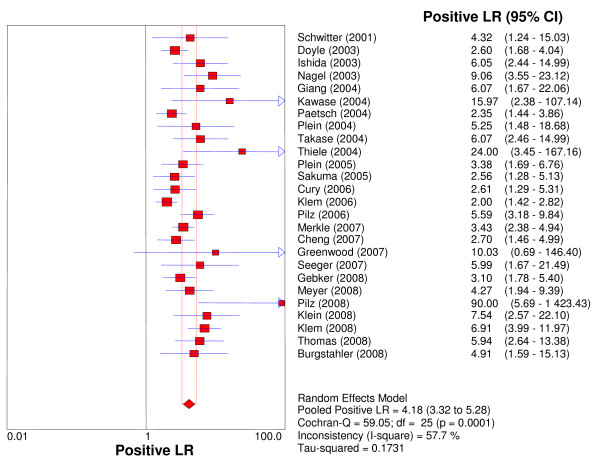
**Forest plot of patient-level positive likelihood ratio of stress perfusion CMR, compared with coronary angiography**.

**Figure 5:**
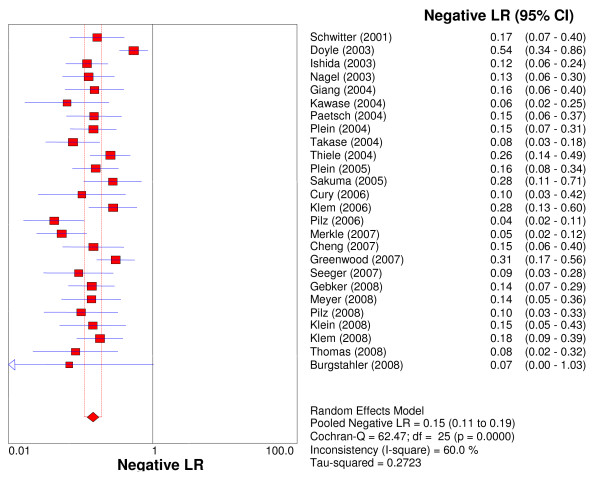
**Forest plot of patient-level negative likelihood ratio of stress perfusion CMR, compared with coronary angiography**.

**Figure 6:**
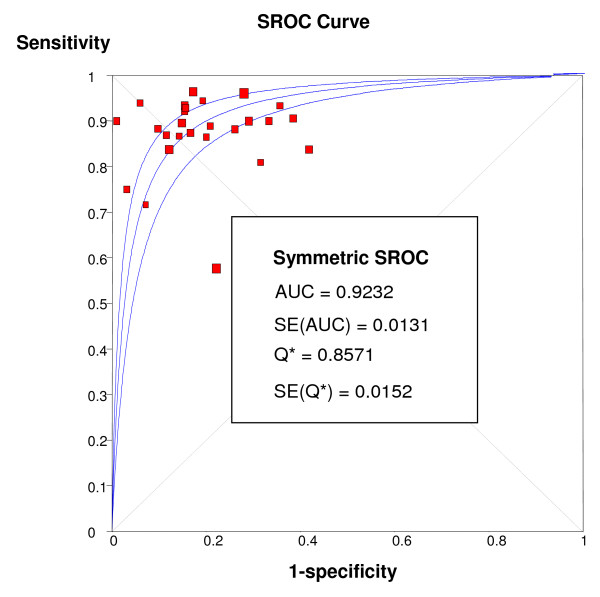
**Plot of symmetric summary receiver operating curve characteristic of stress perfusion CMR, compared with coronary angiography**. The receiver operator characteristic curve provides a graphical display of diagnostic accuracy by plotting 1-specificity in the horizontal axis and sensitivity in the vertical axis. The pertinent area under the curve (AUC) and the Q* statistic (the point where sensitivity and specificity are maximized), both with standard errors (SE), are also included.

Overall per-patient analysis results pooled from 26 studies (2,125 patients) demonstrated a sensitivity of 89% (95% CI: 88-91%), a specificity of 80% (95% CI: 78-83%), a PLR of 4.18 (3.31-5.27), a NLR of 0.15 (95% CI: 0.11-0.20), a DOR of 33.65 (95% CI: 22.09-51.27), and an AUC of 0.92 (Figures 2, 3, 4, 5, 6). Statistical heterogeneity was observed for all relevant diagnostic performance measures. The per-patient prevalence of CAD was 57% (1,205 of 2,125 patients).

**Figure 7:**
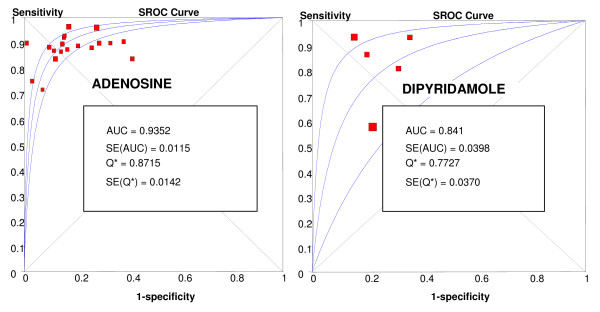
**Plots of symmetric summary receiver operating curve characteristic of stress perfusion CMR, compared with coronary angiography for adenosine and dipyridamole stressors**.

With adenosine as the stressor (20 studies, 1,658 patients) the results were: a sensitivity of 90% (88-92%), a specificity of 81% (78-84%), a PLR of 4.47 (3.39-5.88), a NLR of 0.14 (0.11-0.18), a DOR of 37.17 (25.16-54.91), and an AUC of 0.93. Statistical heterogeneity was observed for all relevant diagnostic performance measures. With dipyridamole as the stressor (5 studies, 417 patients), the results were: a sensitivity of 86% (80-90%), a specificity of 77% (71-82%), a PLR of 2.97 (2.16-4.09), a NLR of 0.20 (0.09-0.45), a DOR of 17.03 (5.56-52.18), and an AUC of 0.84. Statistical heterogeneity was observed for all relevant diagnostic performance measures, except specificity and positive likelihood ratio. ROC curves for stress perfusion CMR performed with adenosine or dipyridamole are shown in Figure 7.

A sensitivity analysis was carried out based on the equipment used (3 Tesla, and 1.5 Tesla MRI). For 3 Tesla (4 studies, 282 patients), results were: a sensitivity of 92% (87-95%), a specificity of 78% (69-85%), a PLR of 3.96 (2.78-5.63), a NLR of 0.12 (0.07-0.20), and a DOR of 35.74 (17.13-74.53). For 1.5 Tesla (23 studies, 1,904 patients), results were: a sensitivity 89% (87-91%), a specificity of 80% (78-83%), a PLR of 4.26 (3.26-5.55), a NLR of 0.15 (0.11-0.20), and a DOR of 34.25 (21.26-55.17).

### Diagnostic performance of stress perfusion CMR: Coronary territory and coronary artery-based analysis

Per-territory results, pooled from 17 studies corresponding to 2,709 coronary territories, demonstrated a sensitivity of 82% (79-84%), a specificity of 84% (82-85%), a PLR of 4.90 (3.66-6.55), a NLR of 0.23 (0.20-0.27), and a DOR of 23.23 (18.33-29.45). At the territory level heterogeneity was significant for all relevant diagnostic performance measures except sensitivity, negative likelihood ratio and diagnostic odd ratios.

Per-artery analysis pooled 8 datasets and demonstrated for left anterior descending artery (LAD), circumflex artery (CX) and right coronary artery (RCA), respectively, sensitivities of 83%, 76% and 78% and specificities of 83%, 87%, and 87%. Statistical heterogeneity was observed for all the performance measurements except sensitivity and negative likelihood ratio for LAD and CX, and diagnostic odds ratio for CX.

## Discussion

This meta-analysis showed stress perfusion CMR to have a high sensitivity (89%) and a moderate specificity (80%) at patient level for the diagnosis of significant obstructive CAD in patients with high prevalence of CAD (57%). We included twelve more studies (on stress perfusion CMR) than the previous meta-analysis by Nandalur et al. [1], which showed a similar diagnostic performance with a pooled sensitivity and specificity of respectively 90% and 81% from 14 perfusion studies. A high false positive rate could have driven the relatively low specificity, and may be due to perfusion defects caused by: [1] dark rim artefacts, the hypo-intensities along the endocardial border of the left ventricular myocardium seen during first-pass transit of a MR contrast medium, thought to be due to a combination of the gadolinium bolus, motion and resolution [45]; [2] the presence of microvascular disease; and [3] spontaneous or therapeutic re-opening of a coronary artery supplying an area of myocardial infarction that has persistent microvascular obstruction [28,32].

Alternatively, because CA detects luminal morphology rather than the functional significance of a stenosis, a false positive CMR results may in fact represent a 'false negative' angiogram in the context of angiographically 'invisible' small vessel disease capable of inducing subendocardial ischemia [40]. This potential source of error could be minimised if the hemodynamic significance of an epicardial coronary artery stenosis were to be determined by the measurement of the fractional flow reserve (FFR) during CA. If validated, this may represent a better reference standard than CA alone. However, although three studies found there to be a good correlation between the performance of stress perfusion CMR and CA with FFR measurement [31,33,35], sufficient data was not present to evaluate its accuracy in this study.

Another point to outline is that for some studies [11,17,19], different decision thresholds to diagnose perfusion CMR as abnormal were appraised: for these studies, the reported sensitivity and specificity could be considered as optimistic because the end points was chosen retrospectively.

In addition, there was a large range of contrast doses used in the individual studies, with the dose of gadolinium administered in the included studies varying by 6-fold, with dose ranging from 0.025 to 0.15 mmole/kg. Although currently there is no consensus regarding the optimal dose and injection rates for perfusion CMR, two multicenter dose-ranging studies have evaluated the impact of contrast dose on the performance of perfusion CMR using a visual analysis [46,47]. In the first, Wolff et al. considered a low dose of 0.05 mmol/kg to be at least as efficacious as any higher dose, and hypothesized that higher doses preformed less well because of the increased likelihood and intensity of artefacts at these doses [46]. However, in the MR-Impact study, Schwitter et al. found better results were obtained using 0.1 mmol/kg [47].

In this meta-analysis, 18 studies were based on stress perfusion CMR alone [10-13,15,17,19-21,24,26,31-33,35,37,39,44], whilst the other 17 included a multi-component examination (cine and/or late gadolinium enhancement (LGE) and/or coronary angiography and/or stress tagging) [14,16,18,22,23,25,27-30,34,36,38,40-43]. In their studies, Plein [22], Cury [28] and Klem [29] evaluated the differences in accuracy based on the sequences evaluated and found that all studies increased accuracy when using a combined analysis. In his study, Klem reported increased specificity (moving from 58% to 87%) when using an algorithm interpretation (including perfusion, cine and LGE).

Having access to data from different sequences (cine, perfusion, and LGE) is especially useful when one component shows a borderline result or is affected by image artefacts. Most of the authors have argued that rest perfusion is an important component because, in combination with late enhancement CMR, it can help distinguish true defects from artefacts on the stress perfusion images.

The fact that the meta-analysis demonstrated a low NLR for stress perfusion CMR suggests that a negative test result may in fact be more clinically useful. This is in keeping with several reports, in different clinical settings, of improved prognosis associated with a normal adenosine stress perfusion CMR scan [48-50]. This meta-analysis also demonstrated adenosine to be superior to dipyridamole as the vasodilating stressor agent. Adenosine may also be safer, with minor side effects of flushing and headache being reported to occur more frequently that any severe adverse effects [51]. Its shorter half life (< 10 s) is an added advantage. Moreover, adenosine has documented safety in the context of non-ST elevation acute coronary syndromes (in a study of 72 patients only one demonstrated intolerance), and in recent ST elevation myocardial infarction [14,22,34].

From this analysis, visual assessment of stress perfusion CMR provided a higher sensitivity but a lower specificity than semi-quantitative assessment. Currently there is no consensus on the superiority of visual over semi-quantitative assessment, or on which method of semi-quantitative assessment should be used. However, the drawbacks of semi-quantitative assessment are that it is more time-consuming, hence not ideal for day-to-day clinical purposes, and the lack of any homogeneous post-processing protocols. Therefore, visual assessment is currently the method most often used in routine clinical practice.

Only 4 studies were performed using 3T CMR, which provides improved resolution [32,38,39,43]. Enhanced sensitivity has been reported [32] and attributed to the higher signal-to-noise and contrast-to-noise ratios permitting improved detection of endocardial perfusion defects. Although most authors argue that the increased prevalence of dark rim artefacts at these higher field strengths (ranging from 8 up to 82%) does not hamper myocardial perfusion analysis [32,39,43], Gebker disagrees and suggests they could limit specificity by increasing false positive rates [38]. In this analysis, 3T CMR was also found to have a decreased specificity, indicating that higher false positive rates may be a real problem. Further studies will be necessary if this controversy is to be resolved.

The results of the per- territory-based analysis showed the anticipated decrease in sensitivity and increase in specificity seen when moving from the level of the patient to that of the coronary territory. Among the 8 studies that performed a coronary-artery level analysis, stress perfusion CMR had a higher sensitivity for detection of significant coronary disease in the LAD artery, compared with the CX and RCA. A possible explanation for this finding may have been the use of a surface radiofrequency coil, which led to lower signal intensities in the more distant inferior and lateral segments.

### Study limitations

Although conventional CA is the established technique for diagnosing significant CAD in routine clinical practice, it remains an imperfect reference standard due to its inability to evaluate the hemodynamic significance of a stenosis.

Substantial inter-study heterogeneity in multiple performance characteristics were observed. Therefore, the pooled performance indices and their interpretation have to be treated with a degree of caution, even though the random-effects model used throughout the analysis should have compensated for this. The observed heterogeneity may have been due to variations in: (i) the image acquisition technique (MR scanner manufacturer, 1.5T or 3T field strengths, pulse sequence, number of slices, contrast dose and rate of infusion); (ii) the interpretation method (visual or semi-quantitative, post-processing techniques); (iii) the patient selection criteria (exclusion or inclusion of patients with prior myocardial infarction, patient populations with differing prevalence of CAD); and (iv) in the definition of significant obstructive CAD (50% or 70%). We noticed, as expected, that studies which performed analysis for 50% and for 70% coronary artery stenosis thresholds, reported an increased sensitivity and a decreased specificity when moving thresholds from 50% to 70% [29,33,36].

These general limitations of stress perfusion CMR could be addressed in future multi-centre studies if standardized imaging protocols, post-processing techniques and patient selection criteria are employed.

## Conclusion

Stress Perfusion CMR has a high sensitivity and moderate specificity for the diagnosis of significant obstructive CAD compared with CA in patients with a high prevalence of the disease.

Future technical developments that increase spatial and temporal resolution whilst reducing artefacts may further improve the diagnostic performance of stress perfusion CMR, and in particular improve its specificity [32]. Currently, however, the low NLR makes stress perfusion CMR particularly accurate and useful in ruling out significant CAD.

## Competing interests

The authors declare that they have no competing interests.

## Authors' contributions

MiH conceived of the study, and participated in its design and coordination and drafted the manuscript. GF, MaH participated in its design and coordination and helped to draft the manuscript. GN, JE, helped to draft the manuscript. RM participated in the design of the study and performed the statistical analysis. All authors read and approved the final manuscript.

## References

[B1] Nandalur KR Dwamena BA Choudhri AF Nandalur MR Carlos RC Diagnostic performance of stress cardiac magnetic resonance imaging in the detection of coronary artery disease J Am Coll Cardiol 2007 50 1343 53 10.1016/j.jacc.2007.06.030 17903634

[B2] Whiting P Rutjes AWS Reitsma JB Bossuyt PMM Kleijnen J The development of QUADAS: a tool for the quality assessment of studies of diagnostic accuracy included in systematic reviews BMC Med Res Methodol 2003 3 25 10.1186/1471-2288-3-25 14606960 PMC305345

[B3] Glas AS Lijmer JG Prins MH Bonsel GJ Bossuyt PM The diagnostic odds ratio: a single indicator of test performance J Clin Epidemio 2003 56 1129 1135 10.1016/S0895-4356(03)00177-X 14615004

[B4] Devillé WL Buntinx F Bouter LM Montori VM de Vet HC Windt DA van der Bezemer PD Conducting systematic reviews of diagnostic studies: didactic guidelines BMC Med Res Methodol 2002 2 9 10.1186/1471-2288-2-9 12097142 PMC117243

[B5] Moses LE Shapiro D Littenberg B Combining independent studies of a diagnostic test into a summary ROC curve: data-analytic approaches and some additional considerations Stat Med 1993 12 1293 1316 8210827 10.1002/sim.4780121403

[B6] Walter SD Properties of the summary receiver operating characteristic (SROC) curve for diagnostic test data Stat Med 2002 21 1237 1256 10.1002/sim.1099 12111876

[B7] Lijmer JG Bossuyt PM Heisterkamp SH Exploring sources of heterogeneity in systematic reviews of diagnostic tests Stat Med 2002 21 1525 1537 10.1002/sim.1185 12111918

[B8] Zamora J Muriel A Abraira V Meta-DiSc for Windows: A Software package for the Meta-analysis of Diagnostic Tests XI Cochrane Colloquium. Barcelona 2003 http://www.hrc.es/investigacion/metadisc.html

[B9] Halligan S Altman DG Evidence-based practice in radiology: step 3 and 4-appraise and apply systematic reviews and meta-analysis Radiology 2007 243 13 27 10.1148/radiol.2431051823 17392245

[B10] Al-Saadi N Nagel E Gross M Bornstedt A Schnackenburg B Klein C Klimek W Oswald H Fleck E Noninvasive detection of myocardial ischemia from perfusion reserve based on cardiovascular magnetic resonance Circulation 2000 101 1379 1383 10736280 10.1161/01.cir.101.12.1379

[B11] Schwitter J Nanz D Kneifel S Bertschinger K Büchi M Knüsel PR Marincek B Lüscher TF von Schulthess GK Assessment of myocardial perfusion in coronary artery disease by magnetic resonance: a comparison with positron emission tomography and coronary angiography Circulation 2001 103 2230 2235 11342469 10.1161/01.cir.103.18.2230

[B12] Ibrahim T Nekolla SG Schreiber K Odaka K Volz S Mehilli J Güthlin M Delius W Schwaiger M Assessment of coronary flow reserve: comparison between contrast-enhanced magnetic resonance imaging and positron emission tomography J Am Coll Cardiol 2002 39 864 870 10.1016/S0735-1097(01)01829-0 11869854

[B13] Sensky PR Samani NJ Reek C Cherryman GR Magnetic resonance perfusion imaging in patients with coronary artery disease: a qualitative approach Int J Cardiovasc Imaging 2002 18 373 383 10.1023/A:1016057821005 12194678

[B14] Chiu CW So NM Lam WW Chan KY Sanderson JE Combined first-pass perfusion and viability study at MR imaging in patients with non-ST segment-elevation acute coronary syndromes: feasibility study Radiology 2003 226 717 722 10.1148/radiol.2263011902 12601212

[B15] Doyle M Fuisz A Kortright E Biederman RW Walsh EG Martin ET Tauxe L Rogers WJ Merz CN Pepine C Sharaf B Pohost GM The impact of myocardial flow reserve on the detection of coronary artery disease by perfusion imaging methods: an NHLBI WISE study J Cardiovasc Magn Reson 2003 5 475 485 10.1081/JCMR-120022263 12882078

[B16] Ishida N Sakuma H Motoyasu M Okinaka T Isaka N Nakano T Takeda K Noninfarcted myocardium: correlation between dynamic first-pass contrast-enhanced myocardial MR imaging and quantitative coronary angiography Radiology 2003 229 209 216 10.1148/radiol.2291021118 12944596

[B17] Nagel E Klein C Paetsch I Hettwer S Schnackenburg B Wegscheider K Fleck E Magnetic resonance perfusion measurements for the noninvasive detection of coronary artery disease Circulation 2003 108 432 437 10.1161/01.CIR.0000080915.35024.A9 12860910

[B18] Bunce NH Reyes E Keegan J Bunce C Davies SW Lorenz CH Pennell DJ Combined coronary and perfusion cardiovascular magnetic resonance for the assessment of coronary artery stenosis J Cardiovasc Magn Reson 2004 6 527 539 10.1081/JCMR-120030580 15137337

[B19] Giang TH Nanz D Coulden R Friedrich M Graves M Al-Saadi N Lüscher TF von Schulthess GK Schwitter J Detection of coronary artery disease by magnetic resonance myocardial perfusion imaging with various contrast medium doses: first European multi-centre experience Eur Heart J 2004 25 1657 1665 10.1016/j.ehj.2004.06.037 15351166

[B20] Kawase Y Nishimoto M Hato K Okajima K Yoshika J Assessment of coronary artery disease with nicorandil stress magnetic resonance imaging Osaka City Med 2004 50 87 94 15819303

[B21] Paetsch I Jahnke C Wahl A Gebker R Neuss M Fleck E Nagel E Comparison of dobutamine stress magnetic resonance, adenosine stress magnetic resonance, and adenosine stress magnetic resonance perfusion Circulation 2004 110 835 842 10.1161/01.CIR.0000138927.00357.FB 15289384

[B22] Plein S Greewood JP Ridgway JP Cranny G Ball SG Sivanathan MU Assessment of non-ST-segment elevation acute coronary syndromes with cardiac magnetic resonance imaging J Am Coll Cardiol 2004 44 2173 2181 10.1016/j.jacc.2004.08.056 15582315

[B23] Takase B Nagata M Kihara T Kameyawa A Noya K Matsui T Ohsuzu F Ishihara M Kurita A Whole-heart dipyridamole stress first-pass myocardial perfusion MRI for the detection of coronary artery disease Jpn Heart J 2004 45 475 486 10.1536/jhj.45.475 15240967

[B24] Thiele H Plein S Breeuwer M Ridgway JP Higgins D Thorley PJ Schuler G Sivananthan MU Color-encoded semiautomatic analysis of multi-slice first-pass magnetic resonance perfusion: comparison to tetrofosmin single photon emission computed tomography perfusion and X-ray angiography Int J Cardiovasc Imaging 2004 20 371 384 10.1023/B:CAIM.0000041938.45383.a4 15765860

[B25] Okuda S Tanimoto A Satoh T Hashimoto J Shinmoto H Higuchi N Nozaki A Kuribayashi S Evaluation of ischemic heart disease on a 1.5 Tesla scanner: combined first-pass perfusion and viability study Radiat Med 2005 23 230 235 16012398

[B26] Plein S Radjenovic A Ridgway JP Barmby D Greenwood JP Ball SG Sivananthan MU Coronary artery disease: myocardial perfusion MR imaging with sensitivity encoding versus conventional angiography Radiology 2005 235 423 430 10.1148/radiol.2352040454 15858084

[B27] Sakuma H Suzawa N Ichikawa Y Makino K Hirano T Kitagawa K Takeda K Diagnostic accuracy of stress first-pass contrast-enhanced myocardial perfusion MRI compared with stress myocardial perfusion scintigraphy AJR Am J Roentgenol 2005 185 95 102 15972407 10.2214/ajr.185.1.01850095

[B28] Cury RC Cattani CA Gabure LA Racy DJ de Gois JM Siebert U Lima SS Brady TJ Diagnostic performance of stress perfusion and delayed-enhancement MR imaging in patients with coronary artery disease Radiology 2006 240 39 45 10.1148/radiol.2401051161 16793971

[B29] Klem I Heitner JF Shah DJ Sketch MH Jr Behar V Weinsaft J Cawley P Parker M Elliott M Judd RM Kim RJ Improved detection of coronary artery disease by stress perfusion cardiovascular magnetic resonance with the use of delayed enhancement infarction imaging J Am Coll Cardiol 2006 47 1630 1638 10.1016/j.jacc.2005.10.074 16631001

[B30] Pilz G Bernhardt P Klos M Ali E Wild M Hofling B Clinical implication of adenosine-stress cardiac magnetic resonance imaging as potential gatekeeper prior to invasive examination in patients with AHA/ACC class II indication for coronary angiography Clin Res Cardiol 2006 95 531 538 10.1007/s00392-006-0422-7 16897145

[B31] Rieber J Huber A Erhard I Mueller S Schweyer M Koenig A Schiele TM Theisen K Siebert U Schoenberg SO Reiser M Klauss V Cardiac magnetic resonance perfusion imaging for the functional assessment of coronary artery disease: a comparison with coronary angiography and fractional flow reserve Eur Heart J 2006 27 1465 1471 10.1093/eurheartj/ehl039 16720685

[B32] Cheng AS Pegg TJ Karamitsos TD Searle N Jerosch-Herold M Choudhury RP Banning AP Neubauer S Robson MD Selvanayagam JB Cardiovascular magnetic resonance perfusion imaging at 3-tesla for the detection of coronary artery disease: a comparison with 1.5-tesla J Am Coll Cardiol 2007 49 2440 2249 10.1016/j.jacc.2007.03.028 17599608

[B33] Costa MA Shoemaker S Futamatsu H Klassen C Angiolillo DJ Nguyen M Siuciak A Gilmore P Zenni MM Guzman L Bass TA Wilke N Quantitative magnetic resonance perfusion imaging detects anatomic and physiologic coronary artery disease as measured by coronary angiography and fractional flow reserve J Am Coll Cardiol 2007 50 514 522 10.1016/j.jacc.2007.04.053 17678734

[B34] Greenwood JP Younger JF Ridgway JP Sivananthan MU Ball SG Plein S Safety and diagnostic accuracy of stress cardiac magnetic resonance imaging vs exercise tolerance testing early after acute ST elevation myocardial infarction Heart 2007 93 1363 1368 10.1136/hrt.2006.106427 17309909 PMC2016919

[B35] Kühl HP Katoh M Buhr C Krombach GA Hoffmann R Rassaf T Neizel M Buecker A Kelm M Comparison of magnetic resonance perfusion imaging versus invasive fractional flow reserve for assessment of the hemodynamic significance of epicardial coronary artery stenosis Am J Cardiol 2007 99 1090 1095 10.1016/j.amjcard.2006.11.061 17437733

[B36] Merkle N Wöhrle J Grebe O Nusser T Kunze M Kestler HA Kochs M Hombach V Assessment of myocardial perfusion for detection of coronary artery stenoses by steady-state, free-precession magnetic resonance first-pass imaging Heart 2007 93 1381 1385 10.1136/hrt.2006.104232 17488772 PMC2016921

[B37] Seeger A Doesch C Klumpp B Kramer U Fenchel M Hoevelborn T Gawaz M Claussen CD May AE Miller S MR stress perfusion for the detection of flow-limiting stenoses in symptomatic patients with known coronary artery disease and history of stent implantation Rofo 2007 179 1068 1073 17879175 10.1055/s-2007-963353

[B38] Gebker R Jahnke C Paetsch I Kelle S Schnackenburg B Fleck E Nagel E Diagnostic performance of myocardial perfusion MR at 3 T in patients with coronary artery disease Radiology 2008 247 57 63 10.1148/radiol.2471070596 18305188

[B39] Meyer C Strach K Thomas D Litt H Nähle CP Tiemann K Schwenger U Schild HH Sommer T High-resolution myocardial stress perfusion at 3 T in patients with suspected coronary artery disease Eur Radiol 2008 18 226 233 10.1007/s00330-007-0746-3 17851665

[B40] Pilz G Klos M Ali E Hoefling B Scheck R Bernhardt P Angiographic correlations of patients with small vessel disease diagnosed by adenosine-stress cardiac magnetic resonance imaging J Cardiovasc Magn Reson 2008 10 8 10.1186/1532-429X-10-8 18275591 PMC2267791

[B41] Klein C Gebker R Kokocinski T Dreysse S Schnackenburg B Fleck E Nagel E Combined magnetic resonance coronary artery imaging, myocardial perfusion and late gadolinium enhancement in patients with suspected coronary artery disease J Cardiovasc Magn Reson 2008 10 45 10.1186/1532-429X-10-45 18928521 PMC2575198

[B42] Klem I Greulich S Heitner JF Kim H Vogelsberg H Kispert EM Ambati SR Bruch C Parker M Judd RM Kim RJ Sechtem U Value of cardiovascular magnetic resonance stress perfusion testing for the detection of coronary artery disease in women J Am Coll Cardiol Img 2008 1 436 45 10.1016/j.jcmg.2008.03.010 19356464

[B43] Thomas D Strach K Meyer C Naehle CP Schaare S Wasmann S Schild HH Sommer T Combined myocardial stress perfusion imaging and myocardial stress tagging for detection of coronary artery disease at 3 Tesla J Cardiovasc Magn Reson 2008 10 59 10.1186/1532-429X-10-59 19094196 PMC2615772

[B44] Burgstahler C Kunze M Gawaz MP Rasche V Wöhrle J Hombach V Merkle N Adenosine stress first pass perfusion for the detection of coronary artery disease in patients with aortic stenosis: a feasibility study Int J Cardiovasc Imaging 2008 24 195 200 10.1007/s10554-007-9236-6 17541724

[B45] Di Bella EVR Parker DL Sinusas AJ On the dark rim artifact in dynamic contrast-enhanced MRI myocardial perfusion studies Magn Reson Med 2005 54 1295 1299 10.1002/mrm.20666 16200553 PMC2377407

[B46] Wolff SD Schwitter J Coulden R Friedrich MG Bluemke DA Biederman RW Martin ET Lansky AJ Kashanian F Foo TK Licato PE Comeau CR Myocardial first-pass perfusion magnetic resonance imaging: a multicenter dose-ranging study Circulation 2004 110 732 737 10.1161/01.CIR.0000138106.84335.62 15289374

[B47] Schwitter J Wacker CM van Rossum AC Lombardi M Al-Saadi N Ahlstrom H Dill T Larsson HB Flamm SD Marquardt M Johansson L MR-Impact: a comparison of perfusion-cardiac magnetic resonance with single-photon emission computed tomography for the detection of coronary artery disease in a multicentre, multivendor, randomized trial Eur Heart J 2008 29 480 489 10.1093/eurheartj/ehm617 18208849

[B48] Ingkanisorn WP Kwong RY Bohme NS Geller NL Rhoads KL Dyke CK Paterson DI Syed MA Aletras AH Arai AE Prognosis of negative adenosine stress magnetic resonance in patients presenting to an emergency department with chest pain J Am Coll Cardiol 2006 47 1427 1432 10.1016/j.jacc.2005.11.059 16580532

[B49] Jahnke C Nagel E Gebker R Kokocinski T Kelle S Manka R Fleck E Paetsch I Prognostic value of cardiac magnetic resonance stress tests. Adenosine stress perfusion and dobutamine stress wall motion imaging Circulation 2007 115 1769 1776 10.1161/CIRCULATIONAHA.106.652016 17353441

[B50] Pilz G Jeske A Klos M Ali E Hoefling B Scheck R Bernhardt P Prognostic value of normal adenosine-stress cardiac magnetic resonance imaging Am J Cardiol 2008 101 1408 1412 10.1016/j.amjcard.2008.01.019 18471450

[B51] Bernhardt P Steffens M Kleinertz K Morell R Budde R Leischik R Krämer A Overhoff U Strohm O Safety of adenosine stress magnetic resonance imaging using a mobile cardiac magnetic resonance system J Cardiovasc Magn Reson 2006 8 475 478 10.1080/10976640600575270 16755834

